# Increased expression of matrix metalloproteinase-10, nerve growth factor and substance P in the painful degenerate intervertebral disc

**DOI:** 10.1186/ar2793

**Published:** 2009-08-20

**Authors:** Stephen M Richardson, Paul Doyle, Ben M Minogue, Kanna Gnanalingham, Judith A Hoyland

**Affiliations:** 1 Tissue Injury and Repair Group, School of Clinical and Laboratory Sciences, Stopford Building, The University of Manchester, Oxford Road, Manchester, M13 9PT, UK; 2 Department of Neurosurgery, Greater Manchester Neuroscience Centre, Salford Royal Foundation Trust, Stott Lane, Salford, M6 8HD, UK

## Abstract

**Introduction:**

Matrix metalloproteinases (MMPs) are known to be involved in the degradation of the nucleus pulposus (NP) during intervertebral disc (IVD) degeneration. This study investigated MMP-10 (stromelysin-2) expression in the NP during IVD degeneration and correlated its expression with pro-inflammatory cytokines and molecules involved in innervation and nociception during degeneration which results in low back pain (LBP).

**Methods:**

Human NP tissue was obtained at postmortem (PM) from patients without a history of back pain and graded as histologically normal or degenerate. Symptomatic degenerate NP samples were also obtained at surgery for LBP. Expression of MMP-10 mRNA and protein was analysed using real-time polymerase chain reaction and immunohistochemistry. Gene expression for pro-inflammatory cytokines interleukin-1 (IL-1) and tumour necrosis factor-alpha (TNF-α), nerve growth factor (NGF) and the pain-associated neuropeptide substance P were also analysed. Correlations between MMP-10 and IL-1, TNF-α and NGF were assessed along with NGF with substance P.

**Results:**

MMP-10 mRNA was significantly increased in surgical degenerate NP when compared to PM normal and PM degenerate samples. MMP-10 protein was also significantly higher in degenerate surgical NP samples compared to PM normal. IL-1 and MMP-10 mRNA demonstrated a significant correlation in surgical degenerate samples, while TNF-α was not correlated with MMP-10 mRNA. NGF was significantly correlated with both MMP-10 and substance P mRNA in surgical degenerate NP samples.

**Conclusions:**

MMP-10 expression is increased in the symptomatic degenerate IVD, where it may contribute to matrix degradation and initiation of nociception. Importantly, this study suggests differences in the pathways involved in matrix degradation between painful and pain-free IVD degeneration.

## Introduction

The human intervertebral disc (IVD) is an avascular and aneural tissue comprising a central gelatinous region (the nucleus pulposus, or NP) surrounded by a fibrous ring of highly organised collagen fibres (the annulus fibrosus, or AF) [1]. The extracellular matrix (ECM) of the NP is rich in type II collagen and proteoglycans, predominantly aggrecan, which produces a highly hydrated matrix capable of withstanding the loads experienced within the spine [2,3]. This ECM is constantly being remodelled in a process driven by the constituent NP cells.

During IVD degeneration, there is an imbalance in the normal homeostatic mechanisms, which favours matrix catabolism and leads to a loss of disc height, coupled with ingrowth of both nerves and blood vessels into both the AF and NP [2,4]. We have previously demonstrated that this ingrowth of nerves into the degenerate IVD is associated with low back pain (LBP) [5]. While LBP is multi-factorial, studies have shown that this debilitating condition affecting around 80% of adults at some stage of life is associated with IVD degeneration in approximately 40% of cases [6]. Indeed, in a recent study by Cheung and colleagues [7], it was shown that there is a significant association of lumber disc degeneration imaged by magnetic resonance imaging with LBP.

A number of studies have demonstrated an increase in expression and activity of a range of matrix-degrading enzymes in IVD degeneration, including the matrix metalloproteinase (MMP) [8-11] and more recently ADAMTS (a disintegrin and metalloproteinase with thrombospondin motifs) families [12-16]. In particular, we have shown that MMP-1, -3, -7, -9 and -13 are involved in matrix catabolism during degeneration [4,12,17]. However, to date, no studies have examined the expression or regulation of MMP-10 in IVD degeneration.

MMP-10 (also known as stromelysin-2) is a member of the stromelysin family of enzymes, along with MMP-3 (stromelysin-1) and MMP-11 (stromelysin-3). This family of enzymes exhibit a wide range of substrate specificities, including both proteoglycans and collagens [18]. In addition to its proteolytic activity, MMP-10 has been demonstrated to be a potent activator of a number of MMP pro-enzymes, including pro-MMP-1, -7, -8, -9 and -13 [19,20]. MMP-10 expression has been identified in human articular chondrocytes isolated from osteoarthritic hip cartilage [19], where the authors also demonstrated its ability to activate pro-MMP-1, -8 and -13, which are key enzymes involved in both cartilage and IVD degradation [12,19,21,22]. MMP-10, along with MMP-3, is also thought to be capable of 'super-activating' collagenases such as MMP-1, with studies demonstrating a significant increase in collagen release (of up to 50%) following the addition of MMP-10 to an IL-1-induced model of cartilage degeneration [19,23,24]. This pivotal role of MMP-10 in multiple pro-enzyme activation is believed to shift the balance of activity in favour of MMP activity over MMP inhibition, with resultant increases in enzymatic activity and increased ECM degradation [24,25].

In articular chondrocytes, MMP-10 expression has been shown to be induced by both interleukin-1 (IL-1) and tumour necrosis factor-alpha (TNF-α), which we have previously shown to be involved in the processes leading to IVD degeneration, particularly IL-1 [19,22]. Chen and colleagues [26] also recently showed that nerve growth factor (NGF) stimulation of PC-12 cells strongly induces MMP-10 gene expression. We have previously identified the expression of the neurotrophin NGF and the pain-associated neuropeptide substance P in the human IVD and demonstrated their regulation in NP cells by IL-1 and TNF-α [27]. NGF is also known to regulate the expression of substance P in sensory neurons [28,29] and intrinsic airway neurons [30], which may lead to increased nociception in painful IVD degeneration.

The aim of the current study was to examine the gene and protein expression of MMP-10 in histologically normal human IVD and compare it with that of both non-painful and painful degenerate IVD. Gene expression of MMP-10 was also correlated with that of the pro-inflammatory cytokines IL-1 and TNF-α as well as NGF to identify potential regulatory mechanisms that may drive MMP-10 production in this tissue. Likewise, gene expression of substance P and its correlation with NGF were assessed to establish any association with nociception in those individuals with LBP.

## Materials and methods

### Tissue samples

Human IVD tissue was obtained at either postmortem (PM) or following surgery, with informed consent from the patient or relatives. Local research ethics approval (North West Research Ethics Committee) was obtained for this work. PM samples of normal and degenerate NP were obtained within 18 hours of donor death. Asymptomatic normal and degenerate discs obtained from donors at PM had no documented clinical history of LBP. Samples of degenerate NP were obtained from patients, diagnosed by magnetic resonance imaging, who underwent disc replacement surgery or spinal fusion to relieve chronic LBP. Patients suffering from classical sciatica were excluded from the study. All samples were obtained and processed as previously described [14].

### Histological grading of nucleus pulposus tissues

To establish histological grade of degeneration, NP samples were fixed in 4% paraformaldehyde/phosphate-buffered saline and processed into paraffin wax. Five-micron sections from the tissue blocks were cut and stained with haematoxylin and eosin, and the degree of morphological degeneration was graded according to previously published criteria [3]. The grading system generates a score of between 0 and 12: a grade of 0 to 3 represents a histologically normal (non-degenerate) disc, grades of 4 to 6 indicate mild degeneration, grades 7 to 9 moderate degeneration and grades 10 to 12 severe degeneration.

### Quantitative real-time polymerase chain reaction

Quantitative real-time polymerase chain reaction (QRT-PCR) was conducted on 5 non-degenerate PM NP samples from 4 individuals (ages 30 to 75 years, mean 56 years), 9 degenerate PM NP samples from 4 individuals (ages 30 to 75, mean 59 years) and 13 surgical degenerate NP samples from 11 individuals (ages 28 to 56 years, mean 39 years). Cells were isolated from each sample as previously reported, and RNA was extracted using Trizol™ (Invitrogen Corporation, Carlsbad, CA, USA) in accordance with the instructions of the manufacturer [14]. RNA was then treated with DNAse using the Turbo DNA-free kit (Ambion, Inc., Austin, TX, USA) to remove any DNA contamination. RNA (500 ng) was then reverse-transcribed using Superscript II (Invitrogen Corporation) in accordance with the instructions of the manufacturer.

QRT-PCR was then conducted on an ABI Prism 7000 sequence detection system (Applied Biosystems, Warrington, UK) to investigate the expression of MMP-10, IL-1β, TNF-α, NGF and substance P in both PM and surgical samples. Glyceraldehyde-3-phosphate dehydrogenase (*GAPDH*) pre-designed amplification reagent (Applied Biosystems) was used as a housekeeping gene to allow normalisation. Pre-optimised primer and FAM-MGB (fluorescein-minor groove binder) probe sets were purchased from Applied Biosystems for MMP-10 (forward primer: CATACCCTGGGTTTTCCTCCAA; reverse primer: GTCCGCTGCAAAGAAGTATGTTTTC; probe: CTGCATCAATTTTCC), IL-1β (forward primer: CGGCCACATTTGGTTCTAAGA; reverse primer: AGGGAAGCGGTTGCTCATC; probe: ACCCTCTGTCATTCG), TNF-α (forward primer: CGAACATCCAACCTTCCCAAC; reverse primer: TGGTGGTCTTGTTGCTTAAAGTTC; probe: CCAATCCCTTTATTACCC), NGF (ABI assay ID: Hs00171458_m1) and substance P (ABI assay ID: Hs00243225_m1). Twenty-microlitre reactions were prepared using TaqMan Universal PCR Master Mix (Applied Biosystems) and 10 ng of each cDNA sample. Reactions were performed in triplicate, and results were analysed using the 2^-ΔCt ^method and presented as relative gene expression normalised to GAPDH [31].

Statistical analysis was performed using the Mann-Whitney *U *test to compare the expression of each different gene between PM normal, PM degenerate and surgical degenerate NP samples. Scatterplots were initially drawn to assess correlations between expression of MMP-10 and IL-1, TNF-α or NGF expression and between NGF and substance P, and then Kendall's rank correlation analysis was used to identify statistically significant correlations.

### Immunohistochemistry for matrix metalloproteinase-10 expression

Immunohistochemsitry for MMP-10 expression was conducted on 4 non-degenerate NP samples from 2 individuals (ages 37 and 47 years, mean 42 years), 5 PM NP samples from 4 individuals with mild degeneration (ages 37 to 61 years, mean 49 years), 4 PM NP samples with moderate degeneration (ages 46 to 78 years, mean 62 years), 2 PM NP samples with severe degeneration (ages 46 years), 10 surgical NP samples with mild degeneration (ages 20 to 74 years, mean 42 years), 8 surgical NP samples with moderate degeneration (ages 33 to 69 years, mean 45 years) and 4 surgical NP samples with severe degeneration (ages 34 to 60 years, mean 49 years).

The immunohistochemistry protocol followed was as previously published [12,14]. No antigen retrieval was required, and a mouse monoclonal primary antibody raised against human MMP-10 (1:500 dilution; Abcam, Cambridge, UK) was used. Human placental samples served as positive controls and negative controls used mouse IgG (Dako, Ely, UK) in place of the primary antibody at equal protein concentrations. Following washes, sections were incubated in a 1:300 dilution of biotinylated goat anti-mouse antiserum (Dako) for 30 minutes at room temperature. Binding of the secondary antibody was disclosed with the streptavidin-biotin complex (Dako) technique with 3,3'-diaminobenzidine tetrahydrochloride solution (Sigma-Aldrich, St. Louis, MO, USA). Sections were counterstained with Mayer's haematoxylin (Raymond A Lamb, Eastbourne, East Sussex, UK), dehydrated and mounted with Pertex.

### Statistical analysis

All slides were visualised by means of a Leica RMDB microscope (Leica Microsystems, Wetzlar, Germany), and images were captured by means of a digital camera and Bioquant Nova image analysis system (Bioquant Image Analysis Corporation, Nashville, TN, USA). The proportions of immunopositive NP cells in each grade grouping (that is, 0 to 3 non-degenerate, 4 to 6 mild degeneration, 7 to 9 moderate degeneration and 10 to 12 severe degeneration) were counted and compared for statistical significance using the Mann-Whitney *U *test. Data were then plotted as mean ± standard error to represent the 95% confidence intervals.

## Results

### Gene expression of matrix metalloproteinase-10 in human nucleus pulposus

**Figure 1:**
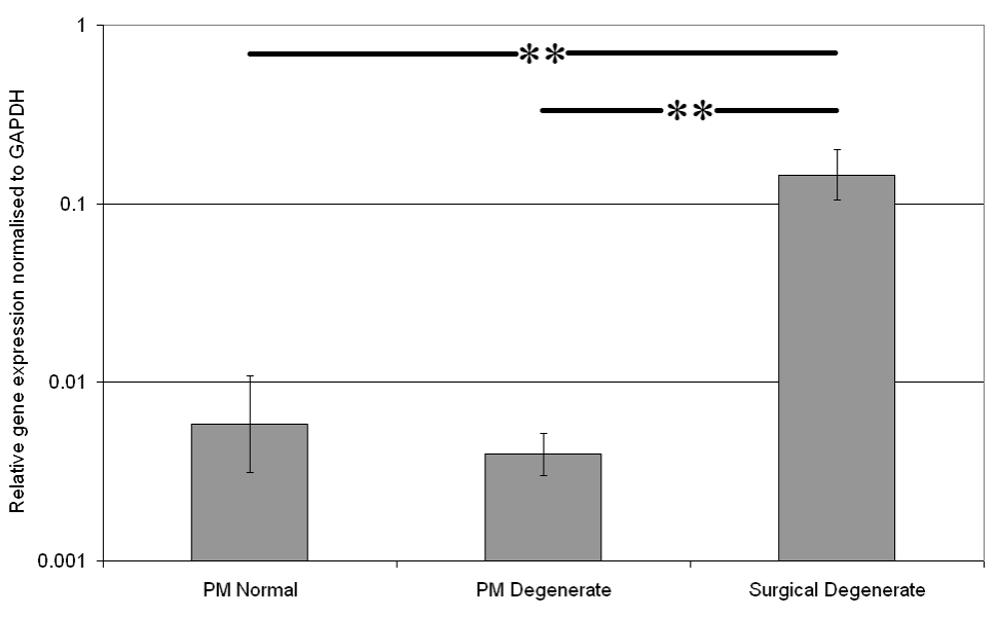
Expression of matrix metalloproteinase-10 in postmortem (PM) normal, PM degenerate and surgical degenerate human intervertebral disc. Relative gene expression was normalised to the housekeeping gene glyceraldehyde-3-phosphate dehydrogenase (*GAPDH*) and plotted on a log scale. **P < 0.01.

QRT-PCR was conducted on RNA from cells extracted from normal and degenerate NP samples obtained at PM and from degenerated NP tissue removed during surgery for LBP. Results demonstrated similar levels of MMP-10 expression in both PM normal and PM degenerate NP samples. However, MMP-10 was significantly higher (*P *< 0.05) in the surgical degenerate samples than in either the PM normal or PM degenerate samples (Figure 1).

### Immunohistochemical localisation of matrix metalloproteinase-10 in human nucleus pulposus

**Figure 2:**
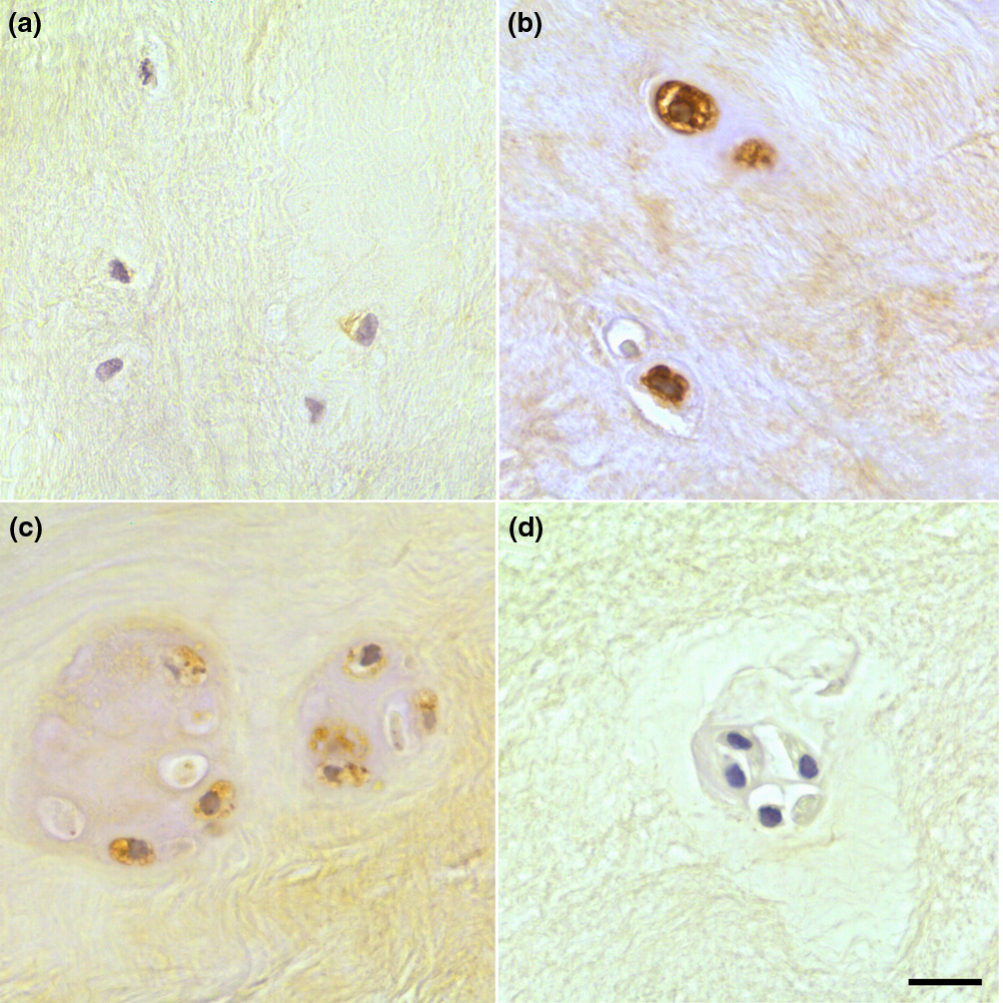
Immunohistochemical localisation of matrix metalloproteinase-10 (MMP-10) in human intervertebral disc. MMP-10 immunopositivity in **(a) **postmortem (PM) normal, **(b) **PM degenerate and **(c) **surgical degenerate samples. An example of an IgG-negative control slide is shown **(d)**. Scale bar = 25 μm.

Immunopositivity was seen for MMP-10 in all samples examined and was evident in NP cells and NP cell clusters (Figure 2). Expression was predominantly localised intracellularly within the cytoplasm of the NP cells. In PM degenerate and surgical degenerate samples, diffuse ECM staining, which was not present in PM normal samples, was observed. No immunopositivity was seen in invading blood vessels or inflammatory cells. Positive controls conducted on placental tissue demonstrated immunopositivity, while all IgG controls were negative.

**Figure 3:**
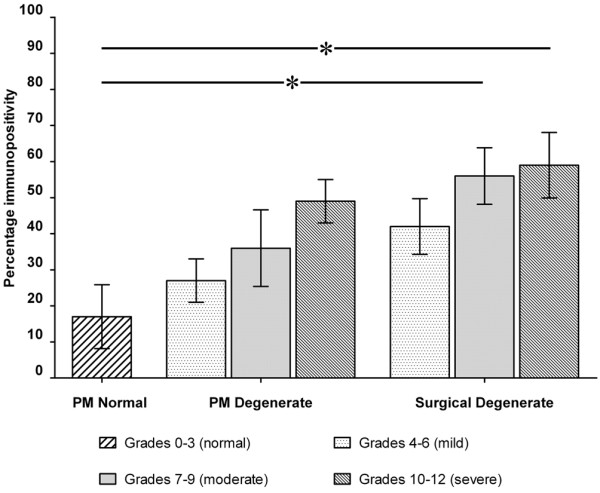
Histogram illustrating the percentage of matrix metalloproteinase-10 immunopositive cells in postmortem (PM) normal, PM degenerate and surgical degenerate nucleus pulposus samples classified according to histological grade of degeneration. Values are mean ± standard error of the mean. *P < 0.05.

While PM normal tissues demonstrated expression in less than 20% of constituent cells, PM degenerate tissues demonstrated increases in the proportion of immunopositive cells with increasing stage of degeneration (Figure 3); however, this did not reach significance at any point. Surgical NP samples again showed increases in the proportion of immunopositive cells with increasing grade. At each grade, the number of immunopositive cells was higher than that seen in PM degenerate tissues of the same grade, although this was not significant. However, surgical NP samples showed significantly higher levels of MMP-10 immunopositivity than PM normal samples at grades 7 to 9 (moderate degeneration) and 10 to 12 (severe degeneration) (*P *< 0.05) (Figure 3).

### Gene expression of interleukin-1 and tumour necrosis factor-alpha and correlation with matrix metalloproteinase-10 in human nucleus pulposus

**Figure 4:**
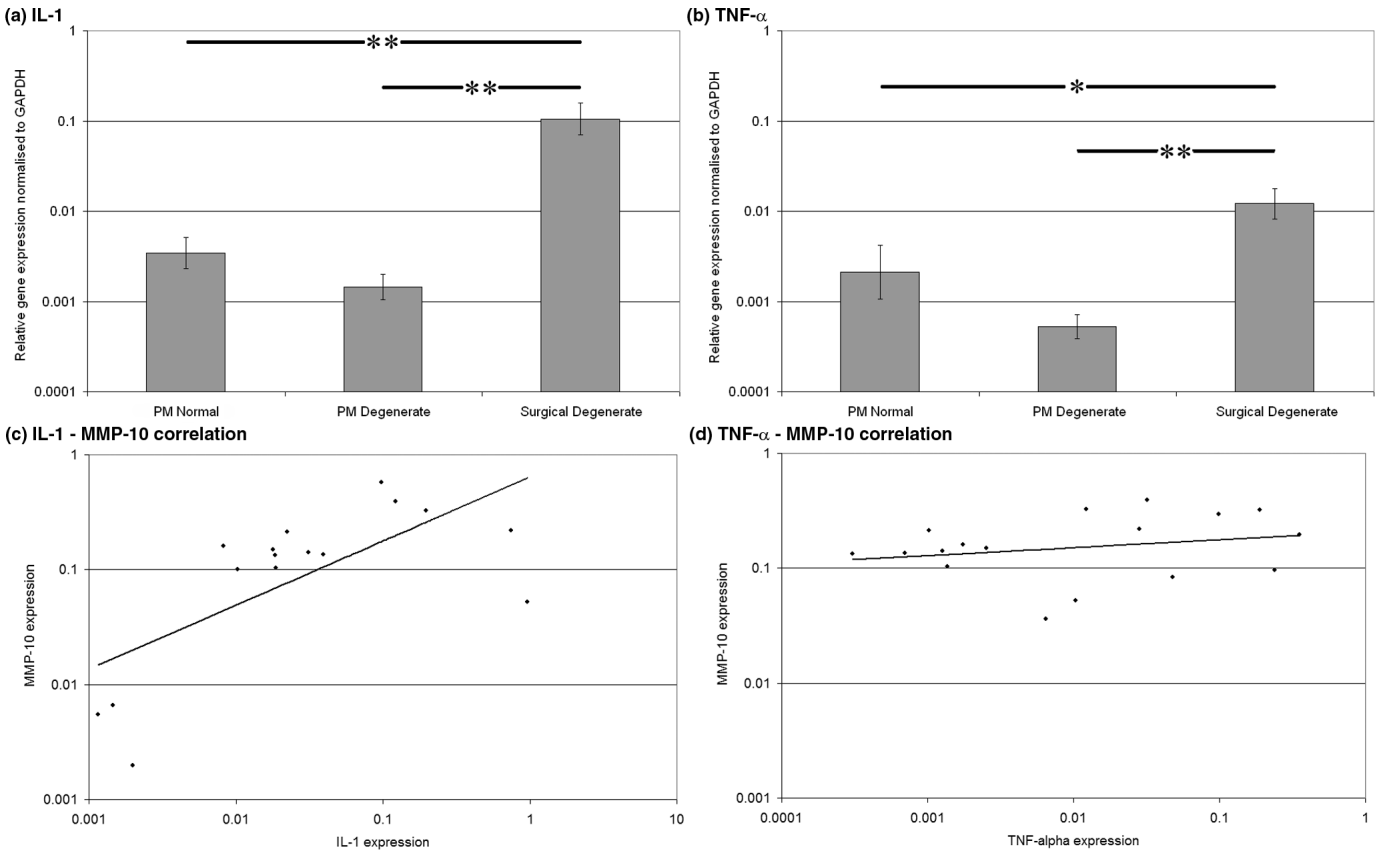
Gene expression data. Histograms illustrating gene expression of **(a) **interleukin-1 (IL-1) and **(b) **tumour necrosis factor-alpha (TNF-α) in postmortem (PM) normal, PM degenerate and surgical degenerate human intervertebral disc. Relative gene expression was normalised to the housekeeping gene glyceraldehyde-3-phosphate dehydrogenase (*GAPDH*) and plotted on a log scale. *P < 0.05; **P < 0.01. Scatterplots illustrating correlations in **(c) **IL-1 versus matrix metalloproteinase-10 (MMP-10) expression and **(d) **TNF-α versus MMP-10 expression in surgical degenerate samples.

No significant differences in the gene expression of either IL-1 or TNF-α between PM normal and PM degenerate samples were observed (Figures 4a and 4b, respectively). However, expressions of both IL-1 and TNF-α in surgical degenerate samples were significantly higher than in either PM normal (*P *< 0.01 and *P *< 0.05, respectively) or PM degenerate (*P *< 0.01 and *P *< 0.01, respectively) samples.

Kendall's rank correlation analysis revealed no significant correlation between IL-1α and MMP-10 in PM degenerate samples (*P *= 0.076) but did reveal a significant positive correlation in surgical degenerate samples (*P *= 0.02) (Figure 4c). However, TNF-α did not show a significant correlation with MMP-10 in either PM degenerate (*P *= 0.49) or surgical degenerate (*P *= 0.31) samples (Figure 4d). No significant correlation could be identified between any of the genes and age of the donors.

### Gene expression and correlation of nerve growth factor and substance P in human nucleus pulposus

**Figure 5:**
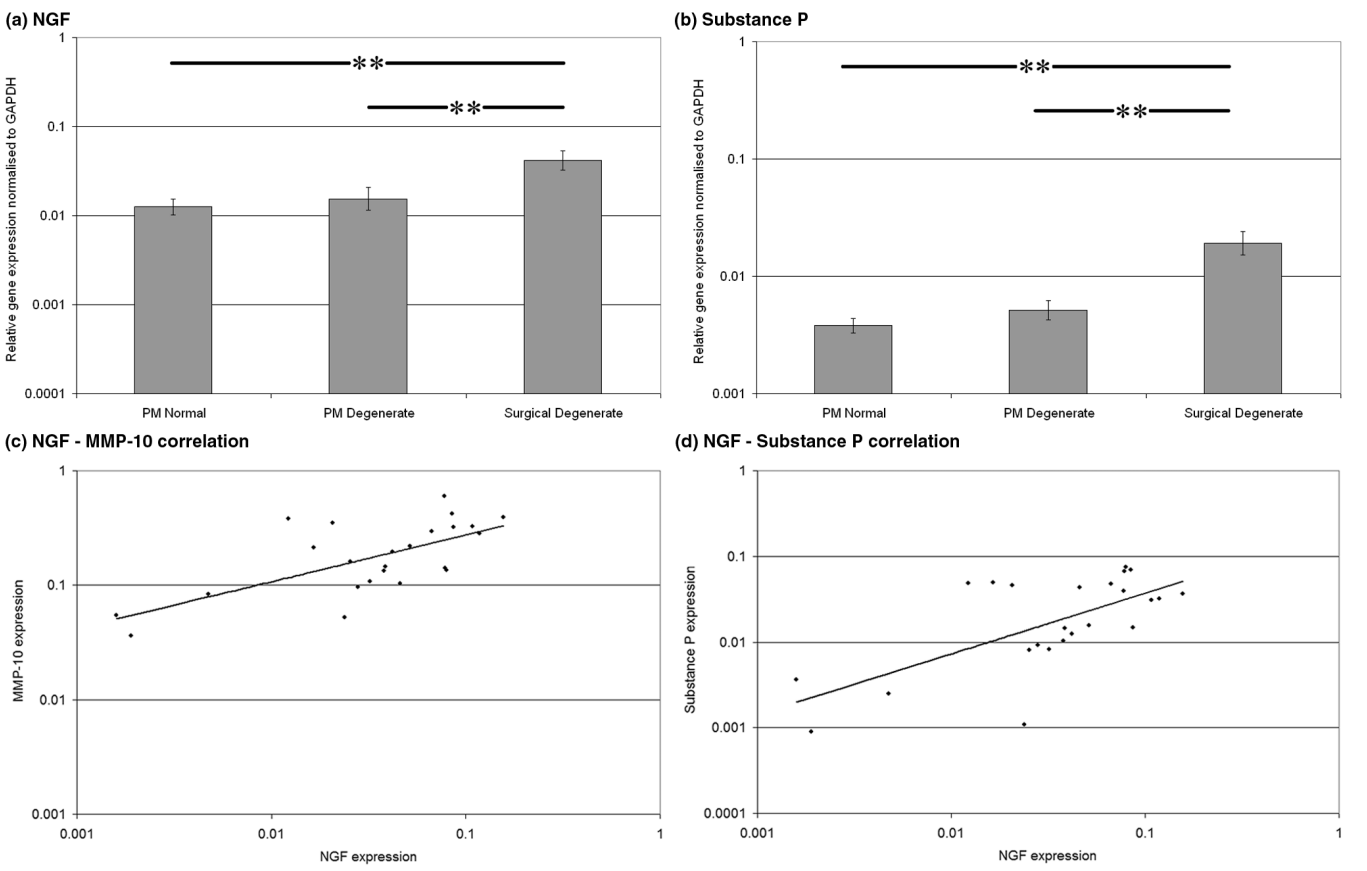
Gene expression data. Histograms illustrating gene expression of **(a) **nerve growth factor (NGF) and **(b) **substance P in postmortem (PM) normal, PM degenerate and surgical degenerate human intervertebral disc. Relative gene expression was normalised to glyceraldehyde-3-phosphate dehydrogenase (*GAPDH*) and plotted on a log scale. **P < 0.01. Scatterplots illustrating correlations in **(c) **NGF versus matrix metalloproteinase-10 (MMP-10) expression and **(d) **NGF versus substance P expression in surgical degenerate samples.

NGF and substance P demonstrated similar levels of expression between PM normal and PM degenerate samples but significantly higher levels of expression in surgical degenerate samples than in either PM normal or PM degenerate samples (*P *< 0.01) (Figures 5a and 5b, respectively). Kendall's rank correlation analysis of NGF and MMP-10 expression data demonstrated no correlation in PM degenerate tissues (*P *= 0.24) but did demonstrate a strong positive correlation in surgical degenerate tissues (*P *< 0.003) (Figure 5c). Analysis of NGF and substance P expression data demonstrated a highly significant positive correlation in surgical degenerate tissues (*P *= 0.001) (Figure 5d) but not in either PM normal or PM degenerate tissues.

## Discussion

The NP of the normal human IVD is an avascular and aneural environment, consisting of chondrocyte-like cells embedded within an ECM rich in proteoglycans and collagens. This matrix is continuously remodelled in a process controlled by the NP cells and closely regulated by anabolic growth factors and catabolic cytokines. In IVD degeneration, there is disregulation in this finely balanced homeostatic matrix turnover mechanism, leading to an increase in catabolic processes over anabolic matrix formation. Over time, this results in the breakdown of matrix until the disc loses both height and function, and in a large proportion of cases, there is innervation and initiation of the pain response which leads to LBP.

Studies have demonstrated the expression of a range of proteolytic enzymes by NP cells, in particular MMP-1, -3, -7, -9 and -13 and ADAMTS-1, -4, -5, -9 and -15 [9,11,12,14,16,17]. These studies have demonstrated significant increases in these enzymes during degeneration and have suggested vital roles for each in the breakdown of the proteoglycan and collagen-rich ECM of the NP.

To our knowledge, this is the first study to focus on the expression on MMP-10 in IVD degeneration. Importantly, we have analysed normal NP obtained at PM and compared it with histologically degenerate NP obtained at PM in patients without a history of LBP and with degenerate NP obtained following surgery for LBP. This enabled us to investigate any differences in gene and protein expression between degenerate NP obtained from individuals who were asymptomatic and those individuals who had similar levels of histological degeneration but who were symptomatic and underwent surgical intervention for their LBP.

Interestingly, in surgical degenerate samples, there were significantly higher levels of MMP-10 gene expression compared with either PM normal or PM degenerate NP samples. Immunohistochemical localisation also demonstrated progressive increases in the number of MMP-10 immunopositive cells within both PM and surgical degenerate samples as disease severity progressed. In the case of surgical NP samples, this increase in immunopositivity over PM normal NP samples was significant in both moderate and severe degeneration. This increase in MMP-10 reflects reported similar changes in a range of MMPs and ADAMTSs during IVD degeneration [12,14], most notably MMP-3, which has a similar structure and substrate specificity [19,20] and demonstrates similar upregulation in degeneration as severity increases [11,12].

Previous studies have demonstrated the catalytic activities of MMP-10. It has wide substrate specificity, including proteoglycans, laminin, fibronectin, gelatin and collagens III, IV, V and IX [18]. In addition to this proteolytic activity, MMP-10 has been shown to play a role in the activation of a number of other members of the MMP family in a range of cell types, including articular chondrocytes [19,20,32]. The activation of MMP by other members of the MMP family is an important factor in MMP regulation and can be a potent influence on ECM breakdown. Barksby and colleagues [19] describe the activation of pro-MMPs by MMP-10 as 'superactivation' as the targets of activation (pro-MMP-1, pro-MMP-8 and pro-MMP-13) have an at least 10-fold higher specific activity than when activated by APMA (4-aminophenylmercuric acetate), trypsin or plasmin [19,25,33]. Such potent activation of these proteins can therefore shift the balance of activity in favour of MMP activity over their inhibitors with resultant ECM breakdown. In addition to MMP-1, MMP-8 and MMP-13, MMP-10 activates MMP-7 and MMP-9. These targets of activation are significant as numerous studies have highlighted the involvement of MMP-1, -7, -9 and -13 in ECM degradation [11,12,15,17]. In particular, two of these MMPs (MMP-7 and MMP-13) target type II collagen and aggrecan and are highly expressed within the NP of the degenerate IVD [12,17], which correlates with our observations regarding MMP-10 localisation to the NP. The wide substrate specificity of MMP-10, coupled with the activity of other MMP-10-activated MMPs, highlights a dual influence of MMP-10 in IVD degeneration.

The results of this study also demonstrate increased expression of both IL-1 and TNF-α in surgical degenerate NP samples over PM normal and PM degenerate samples but no significant differences between the latter PM groups. Interestingly, this study also demonstrates a correlation between IL-1 and MMP-10 expression in the surgical degenerate samples but not in PM normal or PM degenerate samples. Previous studies have shown that IL-1 regulates the expression of MMP-10 in articular chondrocytes [19,32] and this regulation is similar to that shown for MMP-3 in NP cells [22,34]. However, while TNF-α has been demonstrated to regulate MMP-3 in NP cells [35], there is little evidence for its regulation of MMP-10, particularly in chondrocytic cells. Our results also demonstrated no correlation between TNF-α and MMP-10 expression in either PM or surgical degenerate NP samples.

We have previously demonstrated that IL-1 plays an important role in the processes associated with IVD degeneration, in particular in its regulation of MMP expression [22]. IL-1 also regulates expression of NGF in NP cells [27], and the present study has shown significant increases in NGF in surgical degenerate NP samples, which correlates with increases in the expression of MMP-10. The findings also demonstrate a strong correlation between increases in NGF and increases in the pain-associated neuropeptide substance P in surgical degenerate samples but not in either PM normal or PM degenerate samples. Abe and colleagues[36] demonstrated that following stimulation with IL-1 and TNF-α, monolayer NP cells increased expression of NGF, and we have previously shown that when NP cells are cultured in alginate beads, stimulation with IL-1 causes increases in the neurotrophins NGF and BDNF whereas TNF-α causes increases in substance P [27]. The current findings, combined with this previous data, suggest a clear association between pro-inflammatory cytokines IL-1 and TNF-α, the increase of MMP-10, and the expression of NGF and nociception (driven through substance P) in symptomatic IVD degeneration.

These data also support the assumption that IL-1 functions both to enhance the catabolic processes involved in IVD degeneration and to enhance the processes associated with innervation and the pain response that leads to LBP and symptomatic IVD degeneration. Additionally, it is possible that while TNF-α alone does not appear to significantly affect neurotrophin expression, it may be involved in the pain response as it has previously been shown to regulate substance P expression in NP cells [27]. Previous studies have also demonstrated that there is a synergistic effect between IL-1 and TNF-α in the stimulation of NGF by fibroblasts [37]. NGF has previously been shown to stimulate MMP-10 expression [26] and this suggests a possible signalling cascade leading from increases in IL-1 to increases in both NGF and MMP-10 and therefore matrix degradation, innervation and nociception.

Furthermore, our results suggest that there may be differences in the pathways involved in asymptomatic IVD degeneration and symptomatic IVD degeneration that requires surgical intervention for LBP. While in asymptomatic degenerate discs there are clearly increases in MMP and ADAMTS family members, there does not appear to be involvement of MMP-10 or NGF, whereas in symptomatic IVD degeneration, the pathway appears to involve the induced or enhanced expression of both the neurotrophin NGF and MMP-10.

Increases in IL-1 may both directly stimulate the expression of MMP-10 and cause indirect increases in MMP-10 expression through stimulation of NGF expression. The increased expression of MMP-10 may therefore result in increased matrix degradation directly and through 'super-activation' of other MMPs already shown to be increased in IVD degeneration. The increased expression of TNF-α in symptomatic degenerate IVD may also act synergistically to stimulate both MMP-10 and NGF expression whilst also stimulating the expression of substance P and initiating the pain response.

## Conclusions

This study has demonstrated, for the first time, increased MMP-10 expression in the symptomatic degenerate IVD when compared with non-degenerate or asymptomatic degenerate IVD. The correlation of MMP-10 with IL-1 and NGF, combined with the correlation between NGF and substance P in symptomatic degenerate IVDs, suggests differences in the catabolic pathways between painful and pain-free IVD degeneration. While this study focused on gene and protein expression profiling, it emphasises the importance of MMP-10 in symptomatic IVD degeneration and highlights that a more detailed investigation into these pathways, including analysis of enzyme activities, is required to better understand the underlying pathogenesis.

## Abbreviations

ADAMTS: a disintegrin and metalloproteinase with thrombospondin motifs; AF: annulus fibrosus; ECM: extracellular matrix; GAPDH: glyceraldehyde-3-phosphate dehydrogenase; IL-1: interleukin-1; IVD: intervertebral disc; LBP: low back pain; MMP: matrix metalloproteinase; NGF: nerve growth factor; NP: nucleus pulposus; PCR: polymerase chain reaction; PM: postmortem; QRT-PCR: quantitative real-time polymerase chain reaction; TNF-α: tumour necrosis factor-alpha.

## Competing interests

The authors declare that they have no competing interests.

## Authors' contributions

SMR participated in the design of the study, molecular biology work and analysis of results and drafted the manuscript. PD performed the immunohistochemical studies, participated in the molecular studies and performed the statistical analysis. BMM participated in the molecular studies and analysis of results. KG participated in the design of the study and co-wrote the manuscript. JAH conceived of the study, participated in its design and coordination and co-wrote the manuscript. All authors read and approved the final manuscript.
